# Molecular cloning and characteristics of DnaJa1and DnaJb1 in *Coilia nasus:* possible function involved in oogenesis during spawning migration

**DOI:** 10.1186/s12861-019-0187-7

**Published:** 2019-04-25

**Authors:** Xiao-ting Feng, Xue-jun Yang, Jian-jian Ruan, Ya-qi Wang, Yan-feng Zhou, Dong-po Xu, Di-an Fang

**Affiliations:** 1 Scientific Observing and Experimental Station of Fishery Resources and Environment in the Lower Reaches of the Yangtze River, Ministry of Agriculture and Rural Affaris. Freshwater Fisheries Research Center, CAFS, WuXi, 214081 China; 2 Wuxi Fisheries College, Nanjing Agricultural University, Xuejiali 69, Wuxi, 214128 China; 3 National Demonstration Center for Experimental Fisheries Science Education, Shanghai Ocean University, Shanghai, China

**Keywords:** *Coilia nasus*, *DnaJa1and DnaJb1*, Oogenesis, Spawning migration

## Abstract

**Background:**

*Coilia nasus* oogenesis/spawning migration is a well-defined synchronous arrangement process. DnaJs are indispensable molecular chaperones for oogenesis process. However, how DnaJs involved the anadromous spawning migration mechanism is outstanding and plausible.

**Results:**

In this regard, two DnaJs (Cn-DnaJa1 and Cn-DnaJb1) are cloned from the *Coilia nasus’*s ovary. Their structure both contains J domain, G/F domain and ZF domain. Their mRNA transcripts were found extensively expressed in all the sampled tissues and significantly highly in gonads, which probably mean that DnaJs involved in *C. nasus’*s gonad development basal metabolic processes. In the process of spawning migration, *Cn-DnaJa1* and *Cn-DnaJb1* mRNA transcripts were also expressed with significant differences during oogenesis with highest levels in the development phase, and maintaining high levels during the multiplication, mature and spawning phase. Further study showed that the DnaJa1and DnaJb1protein have high distribution in the onset phase and mainly distributed in the oocyte cytoplasm especially during the migration development phase’s.

**Conclusions:**

This experiment study demonstrated that DnaJs participate in reproductive regulation during the spawning migration process in *C. nasus* and possibly play a vital role in the ovary development process. These findings also provided a base knowledge for further molecular mechanism study of spawning migration.

## Background

*Coilia nasus* is widely distributed in the fresh water rivers such as the Changjiang River and coastal waters, which is a kind of anadromous fishery species [1, 2]. Fish anadromous migration is a process preparation for spawning and it is also a highly complex biological event [3]. It is well known that the *C. nasus* spawning migration process was season-depended and temporally based reproductive activity [4]. Sex matured fish usually upstream migrate for a long distance for spawning. During the upstream migration, the fish also undergoes the oogenesis process. When oocytes develop to maturation, fish spawn in the Changjiang River spawning ground. After fertilized, the oosperms float down and hatch along the river side [2, 4]. When up streaming, lots of water stress such as water flow and temperature change will induce numerous adaptive genes such as heat shock proteins (HSPs) up or down regulation to meet the migration behavior need [5–7].

It has been proven that fish oogenesis is a well-defined synchronous arrangement biological process, which can also provide a good biologic model for finding out the regulated mechanism of related genes on oocyte differentiation [8]. During *C. nasus* spawning migration process, there were distinctive phases can be distinguished [8]. It is well known that oogenesis process is regulated by different kinds of HSPs [9]. Various types of HSPs are functioned in a stage-specific and developmentally regulated manner during oogenesis in mouse, rats and humans [10–12].

HSP40 family is an important molecular chaperone in HSPs super family. HSP40 homolog usually contained a highly conserved J domain, which is also named DnaJs [13]. It has a typical N-terminal consensus sequence that facilitates interactions with HSP70 [14]. Although they are moderately conserved, the DnaJ homolog do vary in structures and can be divided into different subtypes [15]. Different types of DnaJs contain different conserved domains, further study found that DnaJ proteins are cofactors for HSP70 [14, 16]. Through its interaction with HSP70, DnaJs are involved in DNA transcription, cell proliferation, signal transduction and other biological processes [1, 9].

Previous studies on ovary transcriptomic analysis in *C. nasus* have shown that DnaJs were up-regulated significantly during spawning migration [17]. Based on this bias, the full length cDNA of *Cn-DnaJa1and DnaJb1* was cloned, tissues depended and temporal mRNA expression patterns in ovaries during different spawning migration phases were investigated; and finally Cn-DnaJa1and DnaJb1 protein during different development phases were clarified. Combination with data from other literatures on fish anadromous migration biology, our findings can support more evidence for the anadromous spawning migration mechanism.

## Results

### Characteristics of DnaJa1and DnaJb1 cDNA

**Fig. 1:**
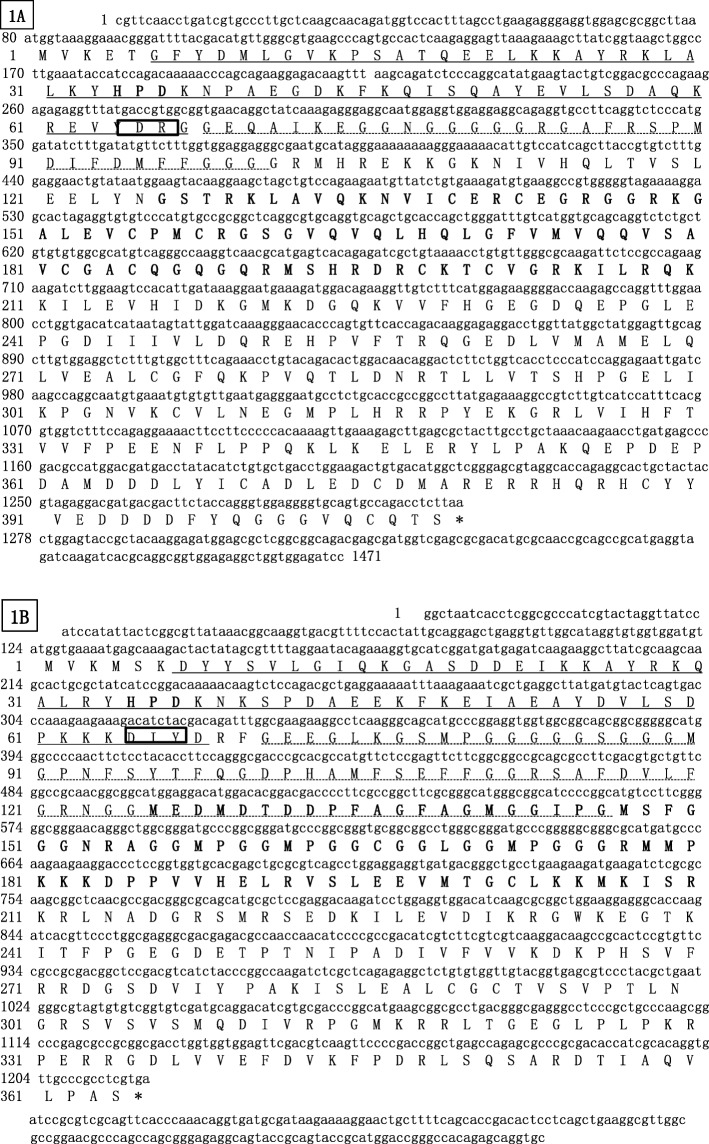
Nucleotide and deduced amino acid sequences of Cn-DnaJa1 and Cn-DnaJb1. The deduced amino acid sequence is shown under the nucleotide sequence. 1**a** is for Cn-DnaJa1 and 1**b** is for Cn-DnaJb1.The termination codon is marked by an asterisk. J domain is showed underlined, HPD motif is boxed, Glycine-rich region profile is signed on the dotted line, Zinc finger CR-type profile (DnaJ CXXCXGXG central domain, 4 repeats) are in bold

Using RACE PCR method, the complete *Cn-DnaJa1* cDNA was 1471 bp in length, which has a 1227 bp open reading frame (ORF), a 79 bp 5′-untranslated region (UTR), and a 165 bp 3′- UTR. The predicted ORF encoded a protein containing 408 amino acids with a calculated molecular weight (Mw) of 45.9 kDa/and a theoretical predicted isoelectric point (pI) of 6.68. Conserved domain analysis identified that DnaJa1 have an N-terminal, a J domain (residues 6–65 aa) containing the conserved histidine proline aspartic acid (HPD) motif (residues 34–36 aa) (Fig. 1a), a Glycine-rich region profile (67–101 aa), a central Zinc Finger domain (126–210 aa) formed by four Zinc Finger CR-type profile (repeats of a CXXCXGXG motif, ZF domain), and a DnaJ C-terminal region (residues 225–335 aa). The Cn-DnaJa1 cDNA and predicted protein sequence had been submitted to GenBank and the accession number is MH748547. The full-length cDNA of *Cn-*DnaJb1 was1376 bp, which containing a predicted ORF of 1095 bp, beginning with a methionine codon at position 124 and ending with a TGA termination codon at position 1204 (Fig. 1b). Its GenBank accession number is MH748548. The encoded 364 amino acid polypeptide had an Mw of 39.4 and a pI of 8.94. As expected, the predicted protein of *Cn-DnaJa1*and *Cn-DnaJb1* had several similar DnaJ family domains, including J domain, HPD domain, G/F domain (glycine- and phenylalanine-rich domain), ZF domain (a cysteine-rich zinc finger domain) and the C domain (a less well-conserved C-terminal) (Fig. 1a & b).

### Homology and phylogenetic analyses

**Fig. 2:**
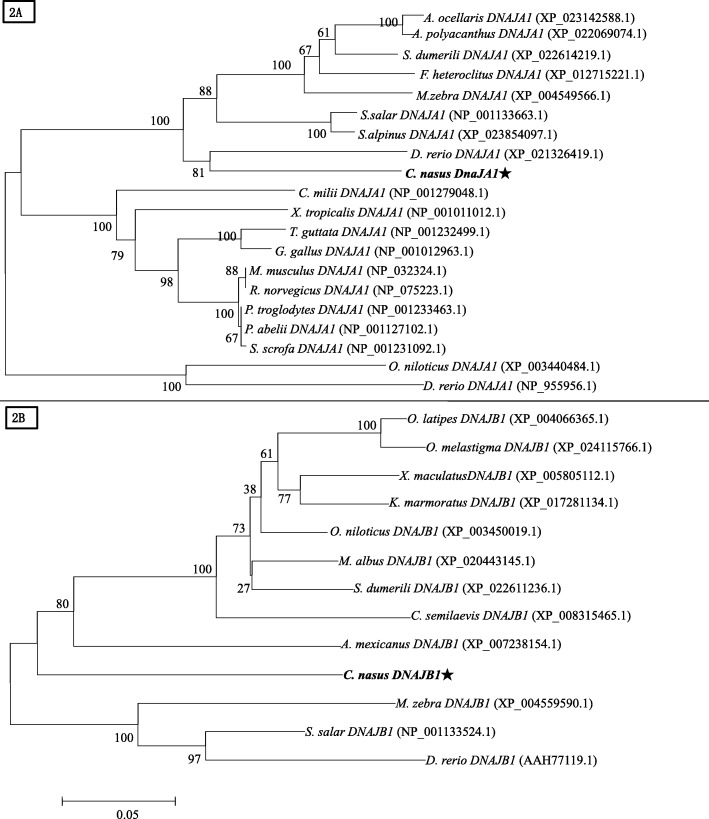
Phylogenetic trees of DnaJa1and DnaJb1family members. Phylogenetic tree constructed by the MEGA 4.0 program by the neighbor-joining (NJ) distance method. 2**a** is for DnaJa1’s and 2**b** for DnaJb1’s. The statistical robustness of the tree was estimated by bootstrapping with 1000 replicates. Bootstrap values were indicated by genetic distance

Phylogenetic trees were constructed by analyzing the amino acid sequences of *C. nasus* DnaJa1and DnaJb1with those from other species, respectively. BLAST analysis suggested that *Cn-DnaJa1* had high sequence homology similarity with *Clupea harengus* (90%), *Salvelinus alpinus* (81%), *Salmo salar* (81%), *Sinocyclocheilus grahami* (80%), *Oncorhynchus mykiss* (80%), *Oryzias latipes* (76%), *Pelodiscus sinensis* (70%), *Otolemur garnettii (*69%), respectively. *Cn-DnaJb1* shared high similarity with that from *Clupea harengus* (81%), *Oncorhynchus mykiss* (72%), *Danio rerio* (72%), *Scleropages formosus* (72%), *Cynoglossus semilaevis* (71%), *Oreochromis niloticus* (69%), *Otolemur garnettii (*69%), *Bos taurus* (64%), respectively. DnaJa1and DnaJb1 protein sequences were obtained from NCBI data base. The produced Neighbor-Joining (NJ) phylogenetic tree indicated that the evolution of DnaJa1and DnaJb1was almost in accordance with the evolution of species (Fig. 2a & b).

### DnaJa1and DnaJb1 mRNA expression patterns

**Fig. 3:**
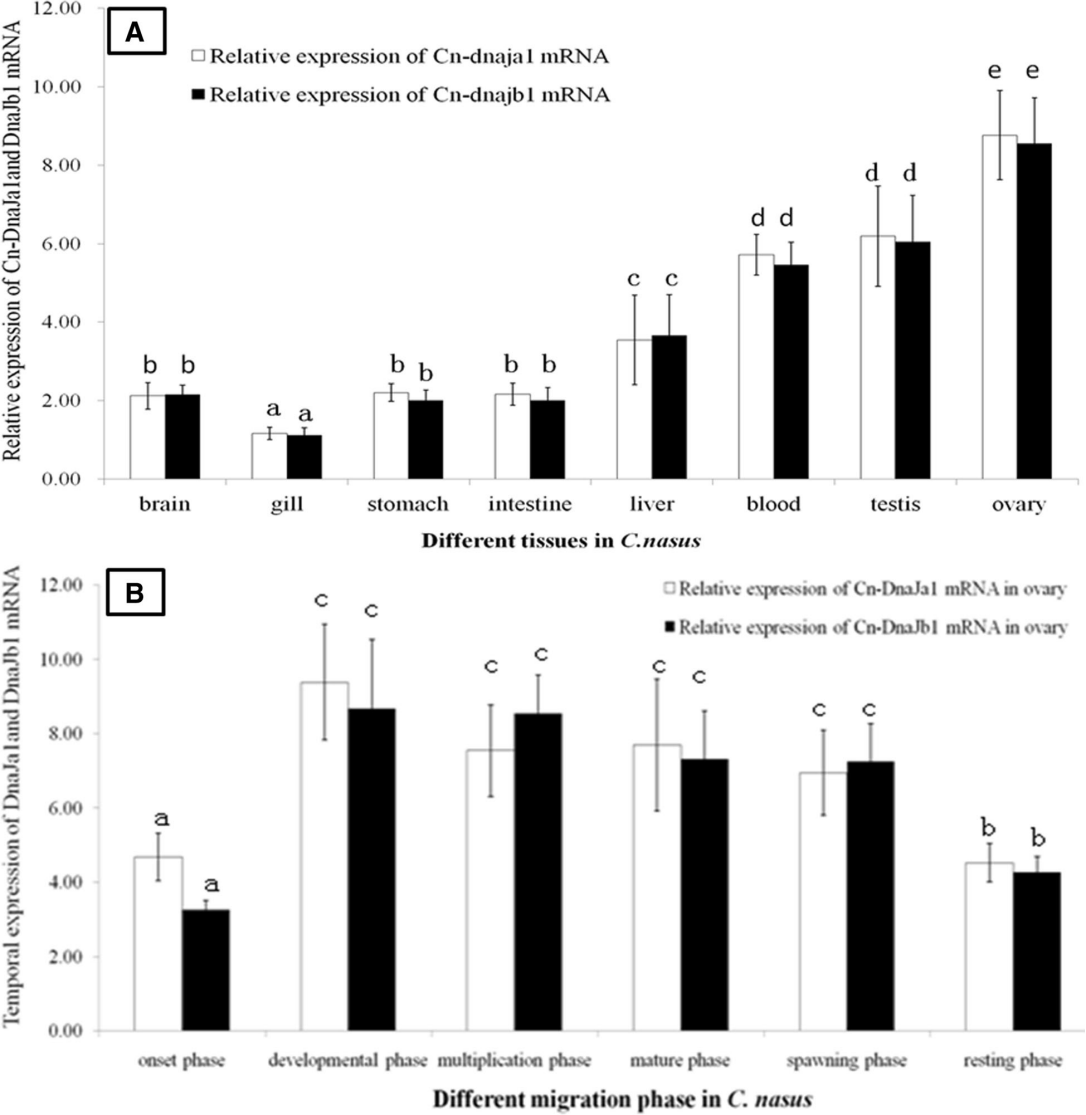
Cn-DnaJa1and Cn-DnaJb1 mRNA expression patterns 3**a** is for Cn-DnaJa1and Cn-DnaJb1 mRNA temporal expression patterns in different tissues. 3**b** is for DnaJa1 and DnaJb1 mRNA temporal expression patterns in different migration phases. Data were expressed as the mean fold difference (mean ± SE, pooled RNA, *n* = 6). Expression values were normalized to those of 18sRNA. Values with the different superscript letters are significantly different (*P* < 0.05, a < b < c < d < e)

*DnaJa1*and *DnaJb1* mRNA transcripts were extensively expressed and testified. Expression levels were found highest in ovary, and higher level in testis, liver and blood than in brain, stomach and intestine, and the gill has the lowest expression level (Fig. 3a). During the spawning migration cycle, *Cn-DnaJa1*and *Cn-DnaJb1* mRNA transcripts expression pattern showed a similar way (Fig. 3b). They were sharply up-regulated to peak level in the development phase, and maintained high levels during the multiplication, mature and spawning phase. Then expression was down-regulated in the resting phase significantly.

### Western blotting (WB) results

**Fig. 4:**
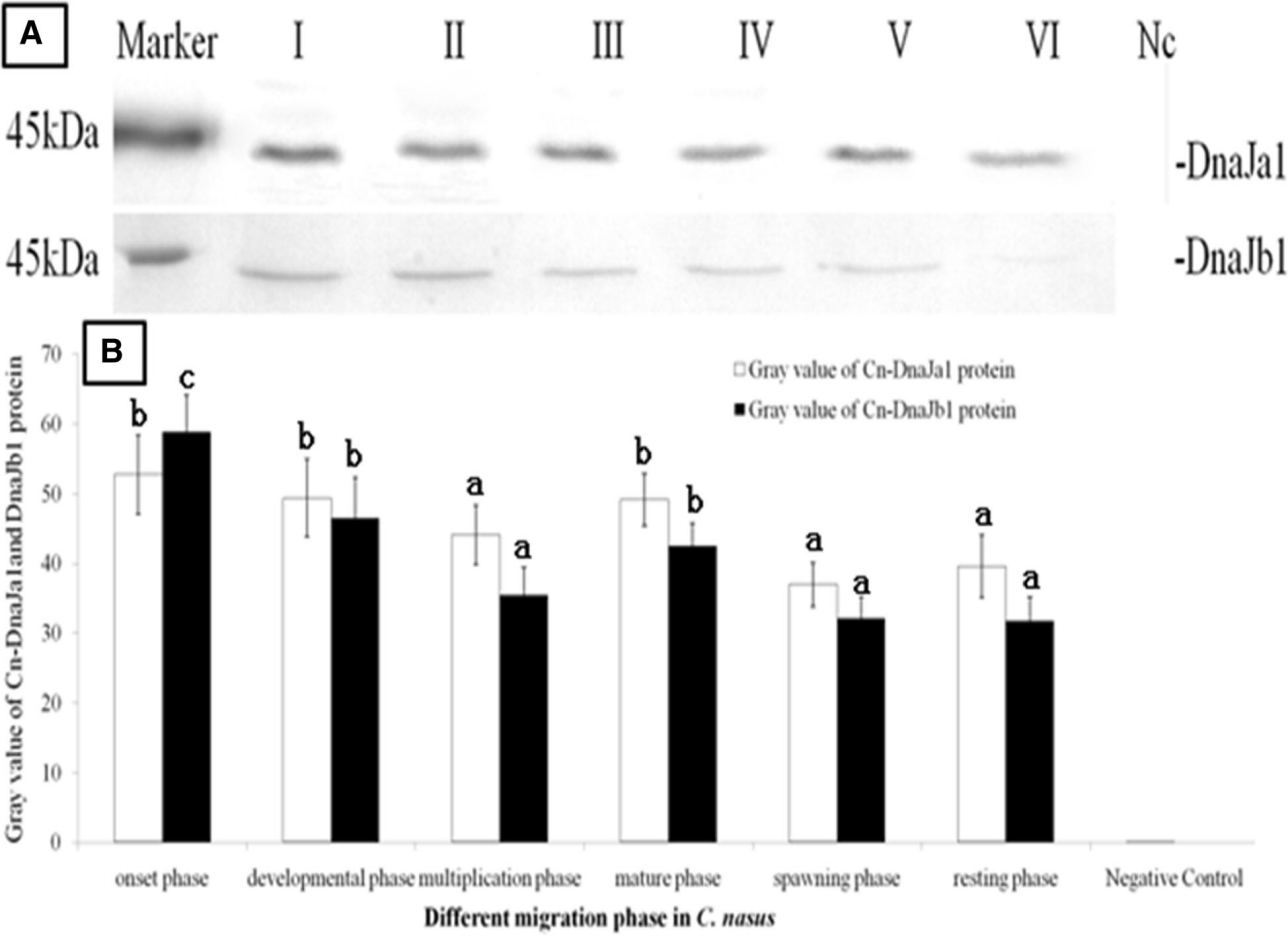
DnaJa1 and DnaJb1 protein expression patterns in different migration phase. Three fish in the mature phase were used for WB. Marker; I: onset phase; II: developmental phase; III: multiplication phase; IV: mature phase; V: spawning phase; VI: resting phase; Nc: Negative Control. 4**a**: DnaJa1and DnaJb1protein expression patterns in different migration phase; 4**b**: The results were semi-quantitated analyzed by ImageJ2x program. Values with the different superscript letters are significantly different (*P* < 0.05, a < b < c)

For WB analysis, the crude protein was extracted from *C. nasus* ovaries in different spawning phase. Anti-DnaJa1, anti-DnaJb1 and anti-serum were also used to recognize the reaction. When the crude protein extracts were transferred to the nitrocellulose membrane and immunoblotted with anti-DnaJa1 and anti-DnaJb1, similar bands were observed on the immunoblot membrane (Fig. 4a). Control serum for the pre-immunized rabbit did not recognize any bands when encountered *C. nasus* ovaries extracts. DnaJa1 protein immunobloted bands were considerately heavier than the DnaJb1’s. These two proteins showed different expression patterns when immunoblotting. DnaJa1 protein increased firstly and then declined, while DnaJb1 protein declined consistently when fish spawning. Between different spawning phases, DnaJa1 and DnaJb1 protein levels both reached the peak in the onset phase and presented declining tendency after that (Fig. 4b).

### Localization of DnaJa1and DnaJb1

**Fig. 5:**
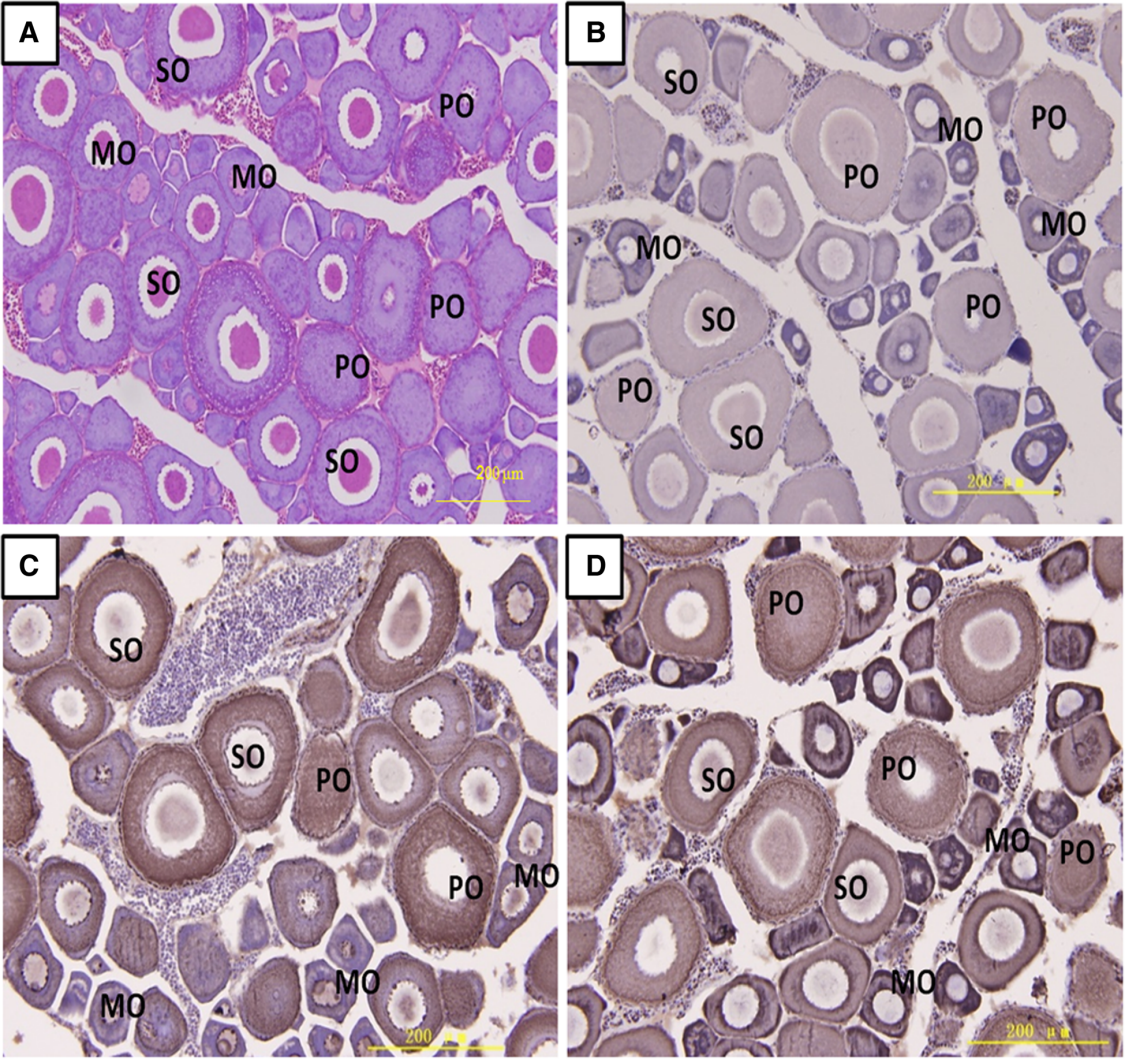
Localization of DnaJa1and DnaJb1 in the mature ovary. Immunohistochemical positive signals of DnaJa1and DnaJb1immunolabeling are shown in brown. **a**: the whole ovary section stained with H&E; **b**: negative control (NC); **c**: different part and developmental phase of ovary for IHC with anti-DnaJa1; **d**: different part and developmental phase of ovary for IHC with anti-DnaJb1. PO: Primary Oocyte, SO: Secondary Oocyte, and MO: Mature Oocyte. Scale bar = 200um

Immunohistochemistry (IHC) method was used to identify the distribution of the DnaJa1and DnaJb1 protein in different types of oocyte. Whole ovary sections were shown in Fig. 5, which were stained with hematoxylin-eosin (H&E, Fig. 5, O1) and immunolabeled with anti-DnaJa1and DnaJb1 (Fig. 5, O3 & O4, counterstained with H&E), respectively. In the normal mature ovary, immunoblotting positive signals were shown in brown both for the DnaJa1 and DnaJb1protein (Fig. 5, O3 & O4). Obviously, the strongest signal for DnaJa1and DnaJb1protein was also detected in the primary oocyte, with the lower signal in the secondary oocyte and there are few observable signals in the mature oocyte (Fig. 5, O3). Interestingly, the DnaJa1 protein was more widely distributed than the DnaJb1’s distribution, and they mainly located in the cytoplasm of different oocyte especially obvious in the development phase (Fig. 5, O3 & O4). There are no positive signals in the negative control, which was incubated with pre-immune rabbit serum (Fig. 5, O2).

## Discussion

In the present study, the complete cDNA sequence and characteristics of *DnaJa1*and *DnaJb1*gene in *C. nasus* were reported. Four conserved domains were also proven to be existed in Cn-DnaJs deductive proteins, which are thought to be involved in substrate binding [13, 16]. By stimulating ATPase activity via this J domain, which can change the conformation of HSP70, and then lead to the folding of substrate polypeptides and the binding to HSP70 [14]. Motif studies on Cn-DnaJa1and DnaJb1 indicated that the highly conserved HPD motif may play a vital role in the activation of HSP70 via the J domain [18]. It is proposed that the HPD domain make special interactions with certain motifs in the ATPase domain of HSP70 through the formation of hydrogen bonds [19]. It was found that these mentioned domains also functioned in Cn-DnaJa1and DnaJb1 structures. These results suggest that Cn-DnaJa1and DnaJb1 probably act as a partner in the HSP70 chaperone complex via a mechanism similar to that observed in *E. coli* DnaJ, which employs the HPD motif as the contact site for HSP70 [20]. In the silkworm, it was detected that there existed two DnaJ proteins with different posttranslational modifications in particular silkworm tissues [21]. In *C. nasus*, two kinds of DnaJ shared high similarity with other fish species sequences and have different post translation modification functions. The results suggested that these structure domains may be essential components in physiology and development biological behavior such as fish spawning migration [19, 22].

As tissue dependent expression patterns analysis showed (Fig. 3a), *Cn*-*DnaJa1*and *Cn*-*DnaJb1*mRNA transcripts were higher expressed in gonads than in other examined tissues. These results indicated that *Cn-DnaJa1* and *Cn-DnaJb1* were potentially synthesized constitutively to support the basic metabolism and development in gonads [12]. Moreover, the expression of *Cn-DnaJa1* and *Cn-DnaJb1* mRNA transcripts was significantly up-regulated in the onset migration phase (Fig. 3b). In our previous transcriptome analysis, DnaJ homologs were the most abundant chaperone at the mRNA level in the normal ovary [17]. During *C. nasus* spawning migration process, fish will encounter lots of stress especially such as water temperature change and rising tide. When *C. nasus* begins migration process, the water temperature is often rising. During fish spawning migration, water temperature is the main inducible factor which induces *C. nasus* oogenesis and spawning [3]. HSPs almost are thermal inducible and ubiquitously existed [11]. It was seen that not only in the development phase but also till the mature phase, both DnaJs temporal expressions are maintained high levels (Fig. 3b). *Cn-DnaJa1* and *Cn-DnaJb1* mRNA transcripts were up-regulated sharply in the onset migration phase and the highest level was observed in the development phase, which is in keeping with the fish oogenesis/migration process. These results may imply that *DnaJa1*and *DnaJb1* may be functioned and essential in mediating anadromous migration initiate [23]. These findings also truly supported that DnaJa1 and DnaJb1 are both constitutive and inducible in the migration process.

When directly exposed to temperature and oxidative stress in the water environment, it has been proven that greater abundance of the DnaJ/HSP70 protein complex could be related to better protection of oocytes in *C. gigas* [24]. Furthermore studies in *R. decussatus* indicated that higher expressions of DnaJ homologs found in good quality oocytes, which might implicate their protective roles in oocytes development [25]. In the present study, WB and IHC results revealed that there were higher DnaJa1and DnaJb1 protein signals in the primary and secondary oocytes (the onset and development phase, Figs. 4 & 5). That is to say, these two proteins are abundant existing in oocyte cells from the beginning of the meiotic stage, and indicated that they are essential for oocyte proliferation and differentiation [11, 26, 27]. These findings are also in close agreement with data from mammals and suggested that Cn-DnaJa1and Cn-DnaJb1proteins are primarily needed on the initial step of migration and gametogenesis in *C. nasus* [11, 27]. Moreover, during the oocyte development and proliferative phase, the very active cytoplasmic protein resembling happened, so that the high level of DnaJa1and DnaJb1 proteins are needed to produce for cell division [28, 29]. Taken together, these findings make biological sense in protein assembling or proceeding, and therefore these proliferating oocytes would require more DnaJa1/ DnaJb1 to guarantee for mature and daughter oocytes for *C. nasus* [8, 11]. Rapid morphological changes are undergoing during oogenesis. Therefore as a result, an increasing high level of DnaJa1and DnaJb1protein in the ovary might lead to high reproductive efficiency [8, 12]. These results indicated that DnaJa1and DnaJb1are not only very sensitive to the oogenesis onset but also necessary to the ovary maturation [29].

DnaJa1 and DnaJb1 reflect highly synthetic activity and protein transport when cytoplasmic accumulation in the development phase [30]. In *Bombyx mori,* BmDnaJ1 protein was mainly found in the cytoplasm of blood cells, which suggested that DnaJ1 is a kind of cytoplasm protein [21]. It is likely that rearrangement of DnaJa1 and DnaJb1 in the oocyte may be required for transport of molecule particles between the germ cytoplasm and cell nucleus [8]. DnaJa1 and DnaJb1are involved in stress-denatured protein folding [16]. When fish up-streaming, they encounter water temperature, tide and water flow stress. To avoid the stress disadvantage, a particular set of HSPs such as DnaJa1 and DnaJb1 will accumulate to protect germ cells against protein denature under these stress during their passage into new development pathways [29, 30].

In conclusion, we characterized DnaJa1 and DnaJb1 molecular structure and their mRNA temporal expression patterns; found that DnaJa1 and DnaJb1 were functional, inducible and essential in the *C. nasus* ovary development and migration process, suggested their compulsory roles in this process. The findings also indicated that DnaJa1 and DnaJb1 are highly expressed in the maintenance of developing oocytes; which implicated greater abundance of the DnaJs protein could better oocyte quality when fish anadromous migration. Our results expanded and supported earlier reports on DnaJs indispensible function to oocyte development.

## Conclusions

In the present study, *Cn-DnaJa1* and *Cn-DnaJb1* complete cDNAs were cloned from the *C. nasus* ovary. Their typical structures and mRNA transcripts were found extensively expressed with significantly higher in gonads. The results proved that Cn-DnaJs involved in *C. nasus’*s gonad development basal metabolic processes. Further temporal expression and their protein distribution analysis indicated that DnaJs participate in reproductive regulation during the spawning migration process in *C. nasus* and possibly play a vital role in the ovary development process. These findings would provide a basic knowledge for further molecular mechanism study of spawning migration.

## Methods

### Fish sampling and tissue collection

Using flow drift net, required fishes were sampled by local fishermen. In short, six reach sections in the Yangtze River were selected for sampling fishes during the anadromous migration period (April to July, 2017) [31]. After fish catching, the sampled fish is dead immediately out of water. All fishes were embedded in ice and dissected to classify the reproductive period and then immediately sampled surgically with the needed tissues (including the blood, brain, gill, liver, testis, ovary, intestine and stomach). After washing by fluidic phosphate-buffered saline (PBS), all tissues were immediately throw into the liquid nitrogen and then transferred to the lab in dry ice boxes storing at − 80 °C for later use. All fish experiment procedures were authorized and approved by the Yangtze River Fish Committee in China.

### Nucleic acid preparation

RNA Extraction kit (Invitrogen, CA, USA) was used to extract total RNA from different sampled tissues. The acquired RNA quality and concentration were identified by agarose gel electrophoresis and the spectrophotometry, respectively. First-strand cDNA was synthesized with reverse transcriptase (Takara Bio Inc., Shiga, Japan) according to the protocol. Target fragments of *Cn-DnaJa1*and *Cn-DnaJb1*were obtained from our constructed transcriptome library. After using BLAST program [32], the target and cloning sequence were verified and selected to obtain the full-length cDNA. After verifing the obtained sequence, the Real-time PCR Kit (Takara, Dalian, China) were used for real-time quantitative RT-PCR (qPCR) analysis. All used primers were designed by Primer Premier 5.0 and synthesized by Wuxi Tianlin Biotech Co. Ltd. (Table 1).

**Table 1:** Sequences of primers used in the present study

Primer Name F—Forward/R—Reverse	DNA-Sequence 5′-3′	Annealing Temperature (°C)	Fragment Size (bp)
Gene-specific Primer pairs for RACE (GsP)			
Gspdnaja1–5′	5’ –TTACCGTGTCTTTGGAGGAACTG − 3′	61.0	-
Gspdnaja1–3′	5’–GCAACTCCATAGCCATAACCAG − 3’	59.2	-
Gspdnajb1–5′	5’- GCGACGAGACGCCAACCAACA − 3’	68.6	-
Gspdnajb1–3’	5’- GGGATGTTGGTTGGCGTCTCGTC − 3’	69.4	-
Primers for RT-qPCR			
Dnaja1-F	5′ –AAAACCCAGCAGAAGGAGACA-3′	58.9	259
Dnaja1-R	5’ –AGTTCCTCCAAAGACACGGTAAG-3’	59.6	
Dnajb1-F	5’-GCGACGAGACGCCAACCAACA-3′	68.6	151
Dnajb1-R	5′-GGGACGCTCACCGTACAACCACA − 3’	68.7	
18sRNA primers			
18sRNA-R	5′- TGATTGGGACTGGGGATTGAA-3′	59.2	232
18sRNA-F	5′- TAGCGACGGGCGGTGTGT-3′	62.4	

### Gene cloning of DnaJa1and DnaJb1

Rapid amplification of cDNA ends (RACE) technology was used to acquire the full length sequence of *DnaJa1* and *DnaJb1* cDNA. Two pairs of gene-specific primers (Gsp) for *DnaJa1and DnaJb1* (GspDnaJa1–5′, GspDnaJa1–3′; GspDnaJb1–5′ and GspDnaJb1–3′; Table 1) were used for their cDNA full-length sequence cloning. Their RACE PCR reactions were performed according to the SMARTer™ RACE cDNA amplification kit protocol (Clontech, Madison, USA). The positive PCR products were sequenced by Wuxi Tianlin Biotech Co. Ltd.

### Analysis of expression patterns

*DnaJa1* and *DnaJb1* mRNA transcripts expression patterns were testified by qPCR method, respectively. Briefly, after acquiring the cloned *DnaJa1* and *DnaJb1* cDNA, primers (DnaJa1-F, DnaJa1-R; DnaJb1-F, DnaJb1-R, Table 1) were designed to produce amplicon of 259 bp and 151 bp, respectively. *18sRNA* gene was selected as the control gene and normalized to samples. The primers of 18sRNA-R and 18sRNA-F were designed to amplify a fragment of 232 bp. All qPCR was performed on a Light Cycler Nano Real-Time PCR System (Roche, USA) in triplicate. The final total volume of each qPCR reaction was 25 μL, which contained 12.5 μL SYBR Premix ExTaq (TaKaRa, Dalian, China), 1.5 μL of diluted cDNA as template, 9 μL of PCR-grade water, and 1 μL of each 10 μM primer. PCR conditions were as follows: 98 °C for 10 s, followed by 35 cycles of 95 °C for 10 s and 56 °C for 30 s. Gene mRNA transcripts expression levels were calculated by the 2^−ΔΔCT^ comparative CT method [33].

### Western blotting

The polyclonal antibody and synthetic peptide were offered by Hua’an Company (Hua’an Biotech Co. Ltd. Hangzhou, China) commercially. Shortly speaking, a synthetic C-terminal peptide (DFYQGGGVQCQTS for anti- DnaJa1; SARDTIAQVLPAS for anti- DnaJb1) conjugated with keyhole limpet hemocyanin (KLH), and then emulsified with complete (for first immunization) and incomplete (for second to fourth) Freund adjuvant; ultimately mixtures were injected into a New Zealand white rabbit at intervals of 2 weeks. After fourth injections, the rabbit was bled, and serum samples were sampled. The antibody titers were verified by enzyme-linked immunosorbent assay (ELISA) method. Under a dissecting microscope, ovaries were dissected from different phases (*n* = 3) in cold PBS. Ovaries were washed in cold 10 mM Tris-HCl (4 °C, pH = 7.4) and grinded, the homogenates were dissolved in electrophoresis sample buffer and run in the polyacrylamide gels. Gels were transferred to nitrocellulose membrane for immunoblotting. The primary antibodies diluted at 1:200 for incubation, and then followed by treatment with goat anti-rabbit IgG (1:80) and determinate with the DAB method. PBS contained 0.5% Bull serum albumin (BSA) was used for a blocking solution. Embedded membranes were observed by gel imaging and analysis system (Bio-Rad Laboratories-Segrate, Italy) and gray value was counted by the software Image J2x 2.1. The average gray value was used to analysis the protein difference expression.

### Immunohistochemistry (IHC)

Ovaries were dissected out from fish in the mature stage and fixed in 0.05 M PBS containing 4% paraformaldehyde at 4 °C for 24 h (n = 3). Frozen sections were conducted for IHC analysis. Briefly, after washing with fluidic PBS three times, samples were dehydrated in different gradient saccharose-PBS solutions (from 30 to 10%) at room temperature overnight, and then embedded in organ optimal cutting temperature (OCT) compound (Sakure, CA, USA). Standard frozen sections (8 μm in thickness) were performed using a Leica microtome (Leica CM1900, Germany). IHC procedure steps were clarified briefly as follows. After washing 10 min in 0.02 M PBS for three times, sections were immersed in 0.01 M citric acid buffer (pH 6.0, containing 0.1% Tween 20), and then autoclaved for 8 min. Then sections were incubated with anti-DnaJa1 (1:200) and anti-DnaJb1 (1:200) at 4 °C overnight. After that, sections were rinsed 10 min with 0.02 M fluidic PBS three times for each washes. Subsequently, sections were incubated with horseradish peroxidase conjugated IgG (goat anti-rabbit) for 30 min, ultimately sections were rinsed with 0.02 M fluidic PBS three times for 15 min. Sections were stained with H & E for good visualization. Immunoreactive signals were identified using diaminobenzidine (DAB) as the substrate. For the negative control, sections were incubated with pre-immune rabbit serum instead of the antibody as the above methods.

### Statistical analysis

Data is shown as mean ± one standard error (SE). Statistical significance was determined by one-way ANOVA and post-hoc Duncan multiple range tests [34]. Significance was set at *P* = 0.05.

## Abbreviations

DAB: Diaminobenzidine
ELISA: Enzyme-linked immunosorbent assay
GsP: Gene-specific Primer
H&E: Hematoxylin-eosin
HSPs: Heat shock proteins
IHC: Immunohistochemistry
KLH: Keyhole limpet hemocyanin
Mw: Molecular weight
OCT: Optimal cutting temperature
ORF: Open reading frame
PBS: Phosphate-buffered saline
PMSF: Phenylmethyl sulfonylfluoride
qPCR: Real-time quantitative RT-PCR
RACE: Rapid amplification of cDNA ends
RT-PCR: Reverse transcriptase
UTR: Untranslated region
WB: Western blotting
ZF domain: Zinc finger domain
